# Dopamine, Noradrenaline and Differences in Sexual Behavior between Roman High and Low Avoidance Male Rats: A Microdialysis Study in the Medial Prefrontal Cortex

**DOI:** 10.3389/fnbeh.2017.00108

**Published:** 2017-06-07

**Authors:** Fabrizio Sanna, Jessica Bratzu, Maria A. Piludu, Maria G. Corda, Maria R. Melis, Osvaldo Giorgi, Antonio Argiolas

**Affiliations:** ^1^Department of Biomedical Sciences, Section of Neuroscience and Clinical Pharmacology, and Centre of Excellence for the Neurobiology of Addictions, University of CagliariCagliari, Italy; ^2^Department of Life and Environmental Sciences, Section of Pharmaceutical, Pharmacological and Nutraceutical Sciences, University of CagliariCagliari, Italy; ^3^Institute of Neuroscience, National Research Council, Cagliari SectionCittadella Universitaria, Cagliari, Italy

**Keywords:** sexual behavior, dopamine, noradrenaline, mPFC, microdialysis, RHA and RLA rats

## Abstract

Roman High- (RHA) and Low-Avoidance (RLA) outbred rats, which differ for a respectively rapid vs. poor acquisition of the active avoidance response in the shuttle-box, display differences in sexual activity when put in the presence of a sexually receptive female rat. Indeed RHA rats show higher levels of sexual motivation and copulatory performance than RLA rats, which persist also after repeated sexual activity. These differences have been correlated to a higher tone of the mesolimbic dopaminergic system of RHA rats vs. RLA rats, revealed by the higher increase of dopamine found in the dialysate obtained from the nucleus accumbens of RHA than RLA rats during sexual activity. This work shows that extracellular dopamine and noradrenaline (NA) also, increase in the dialysate from the medial prefrontal cortex (mPFC) of male RHA and RLA rats put in the presence of an inaccessible female rat and more markedly during direct sexual interaction. Such increases in dopamine (and its main metabolite 3,4-dihydroxyphenylacetic acid, DOPAC) and NA were found in both sexually naïve and experienced animals, but they were higher: (i) in RHA than in RLA rats; and (ii) in sexually experienced RHA and RLA rats than in their naïve counterparts. Finally, the differences in dopamine and NA in the mPFC occurred concomitantly to those in sexual activity, as RHA rats displayed higher levels of sexual motivation and copulatory performance than RLA rats in both the sexually naïve and experienced conditions. These results suggest that a higher dopaminergic tone also occurs in the mPFC, together with an increased noradrenergic tone, which may be involved in the different copulatory patterns found in RHA and RLA rats, as suggested for the mesolimbic dopaminergic system.

## Introduction

The Roman High- (RHA) and Low-Avoidance (RLA) outbred rat lines, originally selected for respectively rapid vs. poor acquisition of the active avoidance response in the shuttle-box (Bignami, 1965; Broadhurst and Bignami, 1965; Driscoll and Bättig, 1982; Fernández-Teruel et al., 2002; Giorgi et al., 2007) exhibit significant differences in sexual activity when put in the presence of a sexually receptive female rat. Indeed, RHA rats show higher levels of sexual motivation than RLA rats, as revealed by the higher number of non contact penile erections shown by these rats when put in the presence of an inaccessible receptive female, and better copulatory performances than RLA rats, as revealed by the significant differences in numerous copulatory parameters found mainly in the first copulatory test between the two rat lines, but also after five copulatory tests, although attenuated (Sanna et al., 2014a). Among the most pronounced differences found between RHA and RLA rats is the percent of rats that initiate mounting and intromitting and that ejaculate in the first copulatory test, 80% of RHA rats against 40% of RLA rats, respectively (Sanna et al., 2014a). A large body of experimental evidence suggests that a functionally different dopaminergic tone is involved in the copulation differences between RHA and RLA rats. In fact, the different copulatory patterns of RHA and RLA rats have been found to be differently modified by apomorphine, a mixed D1/D2 dopamine receptor agonist administered at doses that facilitate sexual behavior, and by haloperidol, a D2 dopamine receptor antagonist, administered at low doses that inhibit sexual behavior (Sanna et al., 2014b). Accordingly, RLA rats have been found to be more sensitive to the facilitation and inhibition of sexual behavior induced by apomorphine and haloperidol, respectively, as demonstrated by the greater modifications induced by the two drugs mainly when administered at the lower doses in different copulatory parameters in RLA rats with respect to RHA rats (Sanna et al., 2014b). Perhaps more important for this study, the different copulatory patterns of RHA and RLA rats are also related to differences in the activity of the mesolimbic dopaminergic neurons, whose activity is well known to increase during sexual behavior (Pfaus et al., 1990; Pleim et al., 1990; Pfaus and Phillips, 1991; Damsma et al., 1992; Wenkstern et al., 1993; Balfour et al., 2004; Pitchers et al., 2010, 2013; Beloate et al., 2016). Indeed, in spite of the fact that sexually naïve and sexually experienced RHA and RLA rats have similar basal values of extracellular dopamine in the dialysate from the nucleus accumbens, the concentrations of extracellular dopamine and 3,4-dihydroxyphenylacetic acid (DOPAC, one of its main metabolites), have been found to increase differentially in the dialysates obtained from the nucleus accumbens shell of naïve and sexually experienced RHA and RLA rats in both the anticipatory and consummatory phases of sexual behavior. The above differences were more marked between sexually naïve RHA and RLA rats, but persisted between sexually experienced RHA and RLA rats, although tending to diminish in these rats, as found with the differences in sexual behavior (Sanna et al., 2015).

The functional role of the increases in dopaminergic activity in the nucleus accumbens seen during sexual activity (either in the appetitive and consummatory phases of sexual behavior) is still matter of debate as well as that seen during feeding that, like sexual activity, has a strong motivational valence. Thus, although dopamine in the nucleus accumbens is involved in motivation and mesolimbic dopaminergic neurons are usually referred as rewarding neurons, in the last 15 years, recent studies support the hypothesis that mesolimbic dopamine is not involved with the primary expression of motivated or rewarding behaviors, but rather with learning and memory of stimulus-reward associations (Agmo et al., 1995; Berridge and Robinson, 1998; Ikemoto and Panksepp, 1999; Pitchers et al., 2013, 2014; Beloate et al., 2016; Salamone et al., 2016). In line with this hypothesis, the blockade of dopamine receptors in the nucleus accumbens or inactivation of dopaminergic neurons in the ventral tegmental area have been recently found unable to alter the expression of appetitive and consummatory aspects of copulatory behavior in male rats (Pitchers et al., 2013, 2014; Beloate et al., 2016).

However, a role of other brain areas containing dopamine in the above differences in sexual behavior between RHA and RLA rats cannot be ruled out. Accordingly, it is well known that dopamine exerts facilitatory effects on the anticipatory and the consummatory phases of sexual behavior in laboratory animals and also in humans not only in the nucleus accumbens (Everitt, 1990; Pfaus et al., 1990; Hull et al., 1991; Pfaus and Everitt, 1995; Melis and Argiolas, 2011), but also in other brain areas, such as the medial preoptic area, the hypothalamus and its nuclei (i.e., paraventricular nucleus (PVN); Pfaus and Phillips, 1991; Argiolas and Melis, 1995, 2005, 2013; Hull et al., 1995, 1999; Melis and Argiolas, 1995; Melis et al., 2003; Succu et al., 2007; Pfaus, 2010). Another area that contains dopamine and may play a role in sexual behavior is the medial prefrontal cortex (mPFC; Fernández-Guasti et al., 1994; Agmo and Villalpando, 1995; Agmo et al., 1995; Hernández-Gonzáles et al., 1998, 2007; Kakeyama et al., 2003; Balfour et al., 2006; Afonso et al., 2007; Davis et al., 2010; Febo, 2011). In fact, in this brain area are found the nerve endings of the mesocortical dopamine neurons, with their cell bodies localized in the ventral tegmental area as mesolimbic dopamine neurons. As for the nucleus accumbens, the exact role of this brain area in sexual behavior is far from being clear. Indeed, lesions of the mPFC are usually found unable to alter sexual behavior of male rats with a sexually receptive female (Fernández-Guasti et al., 1994; Agmo and Villalpando, 1995; Agmo et al., 1995; Hernández-Gonzáles et al., 1998, 2007; Kakeyama et al., 2003; Balfour et al., 2006; Afonso et al., 2007), nor the expression of conditioned place preference for sexual reward (Davis et al., 2010). However mPFC lesions, which did not alter the appearance of conditioned place preference for sexual reward, abolished in the same animals the ability to form conditioned aversion toward sexual activity when paired with aversive stimuli (Davis et al., 2010) and selective cell firing during approaching behaviors of a male rat toward an inaccessible sexually receptive female have been measured in the mPFC of male rats (Febo, 2011). These findings led to suggest that mPFC activation during sexual behavior plays a role in the integration of external and internal information for the execution and control of goal-directed behaviors rather than in the expression of innate responses to natural reinforcers (see Goto and Grace, 2005). Accordingly, together with the nucleus accumbens, the mPFC is part of a complex neural system involved in the modulation of motivated behavior (goal-directed behavior), which requires the integration of cognitive information from the mPFC, emotional information from the amygdala and context-related information from the hippocampus, in the nucleus accumbens (Goto and Grace, 2005).

Experimental evidence suggest that dopamine release in the mPFC is involved in the adaptive regulation of motivated behavior, and a deregulation of these mechanisms is thought to play a role in pathological or maladaptive conditions, such as psychiatric disorders as schizophrenia, attention deficit and hyperactive disorders (ADHD), depression (Dunlop and Nemeroff, 2007; Masana et al., 2011), or substance abuse and gambling behavior (Everitt and Robbins, 2005). Interestingly, the activity of dopamine released in the mPFC may be influenced by noradrenaline (NA), which is present in the mPFC at higher levels than dopamine, and in particular by the NA transporter (NET), which is not only more abundant than the dopamine transporter (DAT) in the mPFC (Carboni et al., 1990, 2006; Gresch et al., 1995; Westernik et al., 1998), but also shows an affinity for dopamine even higher than that for NA (Horn, 1973). Since dopamine and NA often cooperate in many mPFC functions, from working memory and attentional set formation and shifting to reversal learning, response inhibition and response to stress (see Robbins and Arnsten, 2009), this raises the possibility that dopamine, alone or together with NA, in the mPFC may also play a role in the behavioral differences between RHA and RLA rats including those found in sexual behavior.

In order to test this hypothesis, the activity of the mesocortical dopaminergic system and of the noradrenergic system in the mPFC were studied in the two RHA and RLA rat lines by means of intracerebral microdialysis. Briefly, dopamine (and its main metabolite DOPAC) and NA were measured in dialysates obtained from the prelimbic (PrL) and infralimbic (IL) compartments of the mPFC of sexually naïve (e.g., never exposed to a receptive female) and sexually experienced RHA and RLA rats (e.g., which underwent five preliminary copulation tests and show constant levels of copulatory activity) when put in presence of an inaccessible receptive female and during direct sexual interaction by high pressure liquid chromatography coupled with electrochemical detection (HPLC-ECD).

## Materials and Methods

### Animals

Outbred RHA and RLA male rats (*N* = 30 for each line, weighing ≈300 g at the beginning of the experimental work) were all from the colony founded in 1998 at the University of Cagliari, Italy (Giorgi et al., 2007). The procedures used for the selective breeding of the Sardinian colony have been already described in detail (Giorgi et al., 2005).

Ovariectomized stimulus SD female rats (250–300 g at the beginning of the experimental work) used in all the experiments, were obtained from Envigo (San Pietro al Natisone, Italy). Animals were acclimated four per cage (38 cm × 60 cm × 20 cm) to the housing facilities of the Department of Biomedical Sciences of the University of Cagliari for a minimum of 10 days before the starting of the experiments, at 24°C, humidity 60%, reversed 12 h light/dark cycle (lights off from 08:00 h to 20:00 h), water and standard laboratory food *ad libitum*. Animals were daily handled for 1–2 min throughout the habituation period, in order to limit manipulation stress during the experiments; in addition, contact with the animal house maintenance personnel was restricted to a single attendant and bedding in the home cages was never changed either the day before or on the day of the experiments. All the experiments were performed between 10:00 h and 18:00 h. This study was carried out in accordance with the recommendations of the guidelines of the European Communities, Directive of September 22, 2010 (2010/63/EU) and the Italian Legislation (D.L. March 4, 2014, n. 26). The protocol was approved by the Ethical Committee for Animal Experimentation of the University of Cagliari (Authorization No. 361/2016-PR, April 08, 2016 to FS).

### Experimental Groups

Sexually naïve and sexually experienced male RHA and RLA rats were used. Sexually naïve rats were rats never exposed to a sexually receptive-ovariectomized and estradiol + progesterone primed-female; sexually experienced rats were rats which already underwent five consecutive copulation tests of 60 min at intervals of 3 days with a receptive female (Sanna et al., 2014a,b). Females were brought in oestrus by treatment with subcutaneous estradiol benzoate (200 μg/rat in peanut oil) and progesterone (0.5 mg/rat in peanut oil), 48 h and 6 h before the copulation tests, respectively. Oestrus was verified by May-Grunwald-Giemsa coloration and microscopical examination of vaginal smears 1 h before the experiments. In agreement with previous studies (Sanna et al., 2014a,b, 2015) five preliminary copulation tests were found to be sufficient to have male Roman rats of both lines showing constant levels of copulatory activity: e.g., sexually experienced RHA and RLA rats satisfied the criterion of at least one ejaculation achieved in each one of the last two tests (one RHA rat and two RLA rats that did not satisfied this criterion, were discarded in this phase). Two days after these preliminary copulatory tests, sexually experienced Roman rats underwent stereotaxic surgery for the implant of a microdialysis probe in the mPFC as described below (Sanna et al., 2015).

### Microdialysis in the mPFC during Sexual Behavior

The day before microdialysis, sexually naïve or experienced RHA and RLA rats were positioned in a stereotaxic apparatus (Stoelting Co., Wood Dale, IL, USA) under isoflurane anesthesia (1.5%–2%; Harvard Apparatus, Holliston, MA, USA) and implanted with a vertical homemade microdialysis probe (dialysis membrane ≈3 mm of free surface; Melis et al., 2003), and directed unilaterally at the mPFC, PrL and IL compartments (coordinates: 3.0 mm anterior and 0.7 mm lateral to bregma, and 5.5 mm ventral to dura; Paxinos and Watson, 2004). The day of the experiment, the animals were transferred during the dark phase of the cycle to the mating cage (45 cm × 30 cm × 24 cm), which was located in a sound proof room lit by a dim red light and containing inside another small Plexiglas cage (15 cm × 15 cm × 15 cm) with 25 holes (Ø 2 mm) in the vertical walls to allow visual, olfactory and acoustic but not direct interaction. After a habituation period of 2 h, the microdialysis probe was connected to a CMA/100 microinfusion pump (Harvard Apparatus, Holliston, MA, USA) with polyethylene tubing and perfused with a Ringer’s solution (147 mM NaCl, 3 mM KCl and 1.2 mM CaCl_2_, pH 6.5), at a flow rate of 2.5 μl/min. After an equilibration period of 2 h of the perfusion medium with the extracellular fluid, dialysate aliquots of 37.5 μL were collected every 15 min during the experiment in ice-cooled polyethylene tubes for the measurement of the concentration of dopamine, DOPAC and NA, as described below. After the collection of at least four dialysate aliquots, a receptive female rat was introduced into the small cage located inside the mating cage for 30 min. During these 30 min, other two dialysate aliquots were collected. In these conditions male rats cannot interact directly with the female, but show non contact erections (see below). After this period, the small cage was removed, copulation was allowed for 75 min, and other five dialysate aliquots were collected. At the end of this period, the female was then removed from the mating cage and an additional dialysate aliquot was collected (Pfaus and Everitt, 1995; Melis et al., 2003; Sanna et al., 2015). Sexual parameters related to the anticipatory and consummatory phases of sexual behavior were recorded throughout the experiment (see below).

### Sexual Behavior

Several parameters of sexual motivation and copulatory performance related to the anticipatory and consummatory phases of sexual behavior were recorded throughout the experiment by an observer who was not aware of the specific experimental conditions, e.g., who did not know the line and the level of sexual experience of the animals used in that experiment (see below). Briefly, the latency to the first non contact erection (NCPEL, timed from the introduction of the receptive female in the inner small cage) and their frequency (NCPEF, the number of non contact penile erections that occur in the period in which the female is present in the inner cage) were recorded. These pheromone-mediated penile erections, which occur in sexually potent male rats in the presence of an inaccessible receptive female, are considered an important index of sexual arousal (Sachs et al., 1994; Sachs, 2000; Melis et al., 2003). When sexual interaction was allowed, e.g., during copulation, latency to mount and intromit (ML and IL, timed from the removal of the inner small cage until the first mount or the first intromission, respectively); frequency of mounting and intromitting (MF and IF, the number of mounts and intromissions, respectively, in the first series of copulatory activity and during the entire copulation period); latency to ejaculation (EL, timed from the first intromission of the first series until ejaculation); frequency of ejaculation (EF, the total number of ejaculations during the copulation test) and post-ejaculatory interval (PEI, timed from the first ejaculation until the next intromission), were recorded. Further, copulatory efficacy (CE; the number of intromissions of a given series divided by the sum of the number of mounts and of intromissions in the same series) and inter-intromission interval (III; the ratio between the ejaculation latency of a given series and the number of intromissions in that series) were also calculated for the first series of copulatory activity (Sachs and Barfield, 1976; Meisel and Sachs, 1994; Melis et al., 2003; Sanna et al., 2014a,b, 2015).

### Determination of Dopamine, DOPAC and Noradrenaline Concentrations in the Dialysate from the mPFC

Dopamine, DOPAC and NA concentrations were measured in 20 μL of of the same dialysate aliquot from the mPFC by high pressure liquid chromatography (HPLC) coupled to electrochemical detection using a 4011 dual cell (Coulochem II, ESA, Cambridge, MA, USA) as already described (Melis et al., 2003). Detection was performed in reduction mode at +350 and −180 mV. The HPLC was equipped with a Supelcosil C18 column (7.5 cm × 3.0 mm i.d., 3 μm particle size; Supelco, Supelchem, Milan, Italy), eluted with 0.06 M citrate/acetate pH 4.2, containing methanol 20% v/v, 0.1 mM EDTA, 1 μM triethylamine, and 0.03 mM sodium dodecyl sulfate as a mobile phase, at a flow rate of 0.6 mL/min and room temperature. The sensitivity of the assay was 0.125 pg for dopamine, 0.1 pg for DOPAC and 0.2 pg for NA.

### Histology

**Figure 1:**
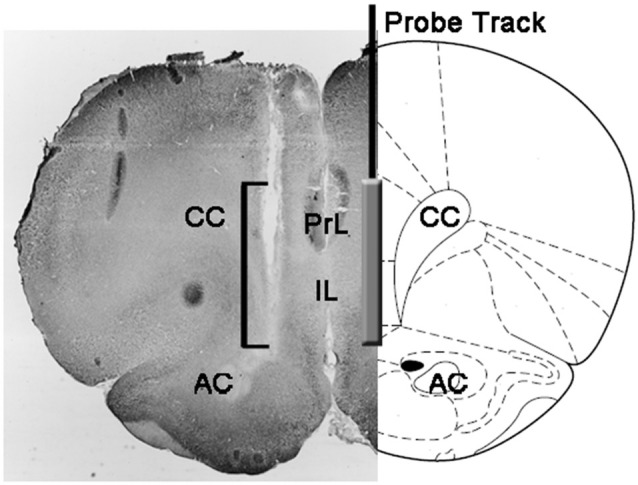
Schematic representation of a coronal section of the rat brain showing the track of the microdialysis probe in the PrL and IL portions of the medial prefrontal cortex (mPFC; Paxinos and Watson, 2004). The square bracket in the micro-photograph indicates the portion of the Neutral Red-stained section showing the active part of the dialyzing membrane of the microdialysis probe. Abbreviations: PrL, prelimbic area; IL, infralimbic area; AC, anterior commissura; CC, corpus callosum.

At the end of the experiments, rats were killed by decapitation, the brains immediately removed from the skull and immersed in 4% aqueous formaldehyde for 12–15 days. After this period, 40 μm coronal brain sections were prepared with a freezing microtome, stained with Neutral Red and inspected on a phase contrast microscope. The position of the tip of the probe was then localized in the mPFC by following the tract of the probe through a series of brain sections (see Figure 1). Only rats found to have the active part of the dialyzing membrane positioned correctly in the PrL and IL compartments of the mPFC were considered for the statistical evaluation of the results (one rat from each experimental group was discarded in this phase).

### Statistics

Statistical analyses of biochemical (dopamine, DOPAC and NA) and behavioral (NCPEL and NCPEF, ML, IL and EL, MF, IF and EF, and PEI) data were conducted either including all experimental subjects (as traditionally done in studies of male rat copulatory behavior) or excluding those subjects who did not copulate to ejaculation during the microdialysis experiment. In the first case, when all animals were included in the analyses, animals that did not display NCPE within the time the female was inaccessible or did not mount or intromit or ejaculate with the available female were assigned the respective full range scores: 1800 s if the male did not display NCPE in the 30 min in which the female was inaccessible; 900 s for ML and IL if the male did not mount or intromit with the available female; 1800 s for EL if the male did not reach ejaculation and 600 s for PEI if the male did not intromit after the first ejaculation. In the second case, those animals that did not copulate to ejaculation were excluded from the analyses. This was done to assess whether the differences in sexual behavior may be correlated with differences in the concentrations of extracellular dopamine, DOPAC and NA in the mPFC of the Roman rat lines during the two phases of sexual activity ruling out possible confusing interferences due to the inclusion of subjects that did not show sexual behavior by assigning them fixed values for the analyzed sexual parameters.

Several statistical analyses were performed with ANOVAs in order to detect and better characterize possible differences between the used experimental conditions. Before performing ANOVAs, data sets of each of the different experimental variables were inspected for homogeneity of variances among the four experimental groups (done with all animals or with rats copulating to ejaculation only) with the Bartlett’s or Levine’s test depending by the case. When significant differences in variances were found, data sets were logarithm transformed (i.e., experimental Y values changed to Log Y values), re-checked for homogeneity of variances and then analyzed by ANOVAs. Briefly, behavioral parameters obtained before (e.g., with the inaccessible female) and during the first series of copulatory activity (from the first mount/intromission to the first mount/intromission after the first ejaculation) of naïve and sexually experienced male RHA and RLA rats during microdialysis were analyzed by two way ANOVAs, by using the rat line and the sexual experience level as between subjects factors (the mean values of the behavioral parameters are reported in Table 1 and the *F* values and significance levels of ANOVA analyses in Table 2).

**Table 1:** Copulatory parameters measured in the first series of copulatory activity (which starts with the first mount/intromission when the female is made accessible to the male and ends after the post ejaculatory interval with the first mount/intrommision of the second series) and non contact penile erections of sexually naïve and experienced RHA and RLA rats.

Behavioral parameters	Naïve		Experienced	
	RHA (*n* = 12)	RLA (*n* = 12)	RHA (*n* = 12)	RLA (*n* = 12)
	*RHA (n =10)*	*RLA (n = 6)*	*RHA (n = 12)*	*RLA (n = 9)*
Non contact penile erection latency (s)	800.6 ± 190.6	1300.6 ± 158.7	570.4 ± 154.0	831.8 ± 152.6
	*600.8 ± 163.3*	*1187.0 ± 247.4*		*946.9 ± 175.2*
Non contact penile erection frequency (s)	2.0 ± 0.5	0.75 ± 0.2	2.8 ± 0.5	1.5 ± 0.3
	*2.4 ± 0.5*	*1.0 ± 0.3*		*1.4 ± 0.4*
Mount latency (s)	297.5 ± 91.1	540.2 ± 106.8	23.7 ± 9.8	296.6 ± 109.0
	*177.0 ± 49.9*	*321.2 ± 106.0*		*95.5 ± 39.8*
Intromission latency (s)	322.4 ± 89.9	562.0 ± 106.9	29.8 ± 11.8	322.1 ± 107.6
	*206.9 ± 54.5*	*355.3 ± 126.4*		*129.5 ± 51.7*
Ejaculation latency (s)	781.5 ± 147.3	1335.3 ± 190.5	355.4 ± 44.8	738.7 ± 197.8
	*577.8 ± 64.5*	*870.6 ± 270.7*		*384.9 ± 95.6*
Mount frequency	5.5 ± 1.6	7.2 ± 2.8	6.8 ± 0.9	6.2 ± 2.4
	*6.6 ± 1.7*	*12.0 ± 4.6*		*8.3 ± 2.9*
Intromission frequency	10.6 ± 1.7	4.0 ± 1.2	13.6 ± 0.9	7.4 ± 2.2
	*12.7 ± 1.1*	*7.5 ± 1.0*		*9.9 ± 2.6*
Ejaculation frequency	3.9 ± 0.6	1.7 ± 0.6	4.3 ± 0.4	2.8 ± 0.6
	*4.64 ± 0.5*	*3.4 ± 0.8*		*3.7 ± 0.5*
Post ejaculatory interval (s)	375.8 ± 41.3	495.5 ± 36.4	339.2 ± 10.5	444.8 ± 35.2
	*330.1 ± 34.1*	*391.0 ± 38.4*		*393.0 ± 30.6*
Copulatory efficacy	0.67 ± 0.03 (*n* = 10)	0.43 ± 0.1 (*n* = 7)	0.69 ± 0.04	0.55 ± 0.07 (*n* = 9)
	*0.67 ± 0.03*	*0.48 ± 0.10*		*0.55 ± 0.07*
Inter Intromission Interval (s)	46.4 ± 5.5 (*n* = 10)	187.2 ± 75.6 (*n* = 7)	26.5 ± 2.9	72.8 ± 29.9 (*n* = 9)
	*46.4 ± 5.5*	*118.4 ± 37.0*		*72.8 ± 29.9*

*For each parameter, the values obtained by considering either all tested animals of the group (including those which were assigned full range scores, *n* = 12, except when differently specified) or the animals of the group which copulate to ejaculation only (written in italics), are reported. One value only is reported for sexually experienced RHA rats, as they all reached ejaculation and the two values are identical. Values are expressed as Mean ± SEM. As no significant interactions between the rat line factor (RHA vs. RLA) and the sexual experience level factor (naïve vs. experienced) were found by analyzing the data with two way ANOVA as shown in Table 2, *post hoc* comparisons were not reported, as explained in the “Materials and Methods”, “Statistics” Subsection*.

**Table 2:** *F* values and significance levels of two-way ANOVA performed on data reported in Table 1 by using the rat line (RHA vs. RLA) and the sexual experience level (naïve vs. experienced) as between subjects factors.

Parameter	*F* values			*df*
	Experience	Line	Experience × Line
Non contact penile erection latency#	2.90	6.99*	0.40	1, 1, 1, 44
	*0.19*	8.53**	*0.17*	*1, 1, 1, 33*
Non contact penile erection frequency	4.96*	13.44***	0.00	1, 1, 1, 44
	*0.99*	10.60**	*0.00*	*1, 1, 1, 33*
Mount latency#	24.04***	13.23***	1.99	1, 1, 1, 44
	23.25***	6.53*	*0.30*	*1, 1, 1, 33*
Intromission latency#	23.18***	12.60***	2.00	1, 1, 1, 44
	19.83***	5.36*	*0.41*	*1, 1, 1, 33*
Ejaculation latency#	11.00**	3.34	0.04	1, 1, 1, 44
	7.46*	*0.00*	*0.19*	*1, 1, 1, 33*
Mount frequency	0.00	0.07	0.31	1, 1, 1, 44
	*0.55*	*2.07*	*0.68*	*1, 1, 1, 33*
Intromission frequency	3.95	15.70***	0.01	1, 1, 1, 44
	*1.04*	7.82**	*0.22*	*1, 1, 1, 33*
Ejaculation frequency	1.64	9.63**	0.35	1, 1, 1, 44
	*0.00*	*2.85*	*0.45*	*1, 1, 1, 33*
Post ejaculatory interval#	0.97	12.11**	0.14	1, 1, 1, 44
	*0.22*	4.66*	*0.12*	*1, 1, 1, 33*
Copulatory efficacy	1.28	9.43**	0.76	1, 1, 1, 34
	*0.58*	7.26*	*0.25*	*1, 1, 1, 33*
Inter intromission interval#	8.93**	10.60**	0.38	1, 1, 1, 34
	6.53*	8.05**	*0.00*	*1, 1, 1, 33*

For each parameter, the values are based on either all tested animals or only on animals that copulated to ejaculation (written in italics). *P < 0.05; **P < 0.01; ***P < 0.001. ^#^Two way ANOVA of the data shown in Table 1 after their logarithm transformation as explained in the “Materials and Methods”, “Statistics” Subsection.

**Figure 2:**
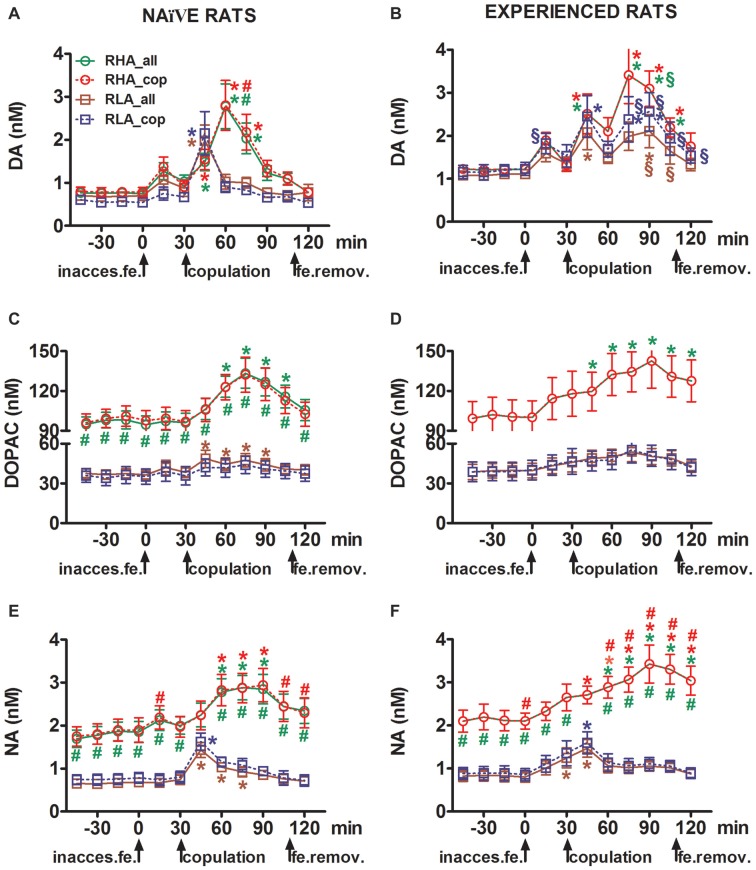
Dopamine (DA), DOPAC and noradrenaline (NA) concentrations in the mPFC dialysates from sexually naïve **(A,C,E)** and experienced RHA and RLA male rats **(B,D,F)** during sexual activity with a receptive female. Sexually naïve (i.e., never exposed before to a sexually receptive female) and sexually experienced (which underwent five copulation tests) of both lines with a microdialysis probe stereotaxically implanted in the mPFC were placed individually into the mating cage. The microdialysis probe was perfused with the dialysis buffer as described in the “Materials and Methods” Section. After the collection of four dialysate aliquots for the determination of basal values, an inaccessible receptive female was then placed inside the small cage of the mating apparatus (time = 0, inaccess.fe). After 30 min, the small cage was removed and copulation was allowed for 75 min (copulation), after which the female was removed from the mating cage (fe.remov). During the experiment, non contact erections and copulatory parameters were measured, and dialysate aliquots collected every 15 min and analyzed for dopamine, DOPAC and NA as described in the “Materials and Methods” Section. Values are means ± SEM of the values obtained by all 12 rats per group (RHA_all = solid green lines, RLA_all = solid brown lines) or those that copulated to ejaculation only (RHA_cop = dashed red lines; RLA_cop = dashed blue lines). In experienced RHA rats all animals reached copulation and the values are identical. *P < 0.05 with respect to basal values (no female) of the group (green for RHA_all; red for RHA_cop, brown for RLA_all, blue for RLA_cop); ^#^*P* < 0.05 with respect to the corresponding values of the RLA group (red, RHA_all vs. RLA_all; green, RHA_cop vs. RLA_cop); ^§^*P* < 0.05 with respect to the time-matched values of the sexually naïve rats (green, experienced RHA_all vs. naïve RHA_all; red, experienced RHA_cop vs. naïve RHA_cop; brown, experienced RLA_all vs. naïve RLA_all; blue, experienced RLA_cop vs. naïve RLA_cop) (two-way ANOVAs done on the shown data after their logarithmic transformation, as explained in “Materials and Methods”, “Statistics” Subsection, followed by Tukey’s HSD tests).

In addition, an overall analysis of the data obtained from each rat during microdialysis was done by calculating first the AUCs obtained by plotting the values of the concentrations of dopamine, DOPAC or NA or the number of non contact erections, mounts, intromissions and ejaculations vs. time (starting 1 h after the introduction of the male rat in the mating cage to the end of the experiment −180 min divided in fractions of 15 min for the neurochemical values- or at the time when the female was introduced in the mating cage for the behavioral parameters) and then by comparing the calculated values by two way ANOVAs by using the rat line and the sexual experience level as between subjects factors (mean values of the AUCs of neurochemical and behavioral parameters are reported in Table 4 and the *F* values and significance levels of ANOVA analyses in Table 5). Finally, a more detailed point to point analysis of each set of data (i.e., the values of the concentrations of dopamine, DOPAC, NA and the numbers of non contact erections, mounts, intromissions, and ejaculations vs. time) was performed by factorial ANOVAs for repeated measures, by using the rat line and the level of sexual experience as between subject factors and time (i.e., dialysate fractions) as within subjects factor (the *F* values and significance levels of these ANOVA analyses are reported in Table 6). Similar analyses were conducted also considering only basal values of neurochemical parameters (last four dialysate aliquots collected before the introduction in the mating cage of the receptive female), whose mean values are reported in Table 3. As first, but not second order interactions were found when performing the general factorial ANOVAs for repeated measures, in particular Line × Time and Experience × Time interactions for neurochemical parameters (see Table 6), two-way ANOVAs with the line or the level of sexual experience as between subjects factor and time as within subject factor were performed on these sets of data by directly comparing sexually naïve or experienced RHA vs. RLA rats, or sexually experienced vs. naïve RHA rats or sexually experienced vs. naïve RLA rats. The results of the *post hoc* pair wise contrasts performed by using the Tukey’s HSD test on significant interactions revealed by these two-way ANOVAs are reported in Figure 2. In all the other cases, *post hoc* comparisons were not reported, as ANOVAs failed to reveal any significant interaction between the rat line, the sexual experience level and the time. Statistical analyses were all carried out with Graph Pad 5 (PRISM, San Diego, CA, USA) and STATISTICA 12 (Statsoft, Tulsa, OK, USA) with the significance level set at *P* < 0.05.

**Table 3:** Basal dopamine (DA), 3,4-Dihydroxyphenylacetic acid (DOPAC) and noradrenaline (NA) concentrations (nM) in the dialysate from the medial prefrontal cortex (mPFC) of sexually naïve and experienced RHA and RLA rats.

	Naïve		Experienced	
	RHA (*n* = 12)	RLA (*n* = 12)	RHA (*n* = 12)	RLA (*n* = 12)
	*RHA (n = 10)*	*RLA (n = 6)*	*RHA (n = 12)*	*RLA (n = 9)*
DA	0.76 ± 0.07	0.69 ± 0.08	1.22 ± 0.08	1.09 ± 0.09
	*0.79 ± 0.08*	*0.56 ± 0.07*		*1.18 ± 0.15*
DOPAC	96.35 ± 6.08	37.03 ± 3.59	100.46 ± 12.77	39.43 ± 5.44
	*98.41 ± 7.16*	*35.34 ± 5.74*		*39.20 ± 7.15*
NA	1.79 ± 0.21	0.66 ± 0.09	2.12 ± 0.24	0.82 ± 0.12
	*1.83 ± 0.24*	*0.75 ± 0.12*		*0.87 ± 0.15*

*Data represent the average of the last four dialysate aliquots collected before the introduction of the receptive female in the mating apparatus and are expressed as Mean ± SEM. Statistical analyses of these data are reported in the “Results” Section. The values are based on either all tested animals or only on animals that copulated to ejaculation (written in italics). All RHA-experienced rats copulated to ejaculation, hence one value is reported*.

**Table 4:** Overall assessment of the differences in DA, DOPAC and NA concentrations and in sexual behavior between sexually naïve and experienced RHA and RLA rats by analysis of the averaged AUCs obtained from the results shown in Figures 2 and 3.

	Naïve		Experienced	
	RHA (*n* = 12)	RLA (*n* = 12)	RHA (*n* = 12)	RLA (*n* = 12)
	*RHA (n = 10)*	*RLA (n = 6)*	*RHA (n = 12)*	*RLA (n = 9)*
DA	209.46 ± 17.6	153.53 ± 17.8	324.98 ± 30.5	251.50 ± 23.3
	*215.73 ± 20.1*	*132.77 ± 11.6*		*289.42 ± 29.9*
DOPAC	17842 ± 1209	6826 ± 687	19615 ± 2412	7517 ± 922
	*17872 ± 1438*	*6366 ± 1025*		*7460 ± 1217*
NA	372.3 ± 39.0	136.6 ± 14.0	440.2 ± 40.1	167.8 ± 21.8
	*376.3 ± 45.6*	*152.0 ± 14.0*		*178.9 ± 26.9*
Non contact penile erections frequency	15.0 ± 3.5	5.6 ± 1.6	21.0 ± 2.7	11.2 ± 2.1
	*18.0 ± 3.5*	*7.5 ± 2.6*		*10.8 ± 2.7*
Mount frequency	324.7 ± 91.2	180.7 ± 57.6	383.2 ± 62.1	231.7 ± 58.3
	*389.7 ± 96.9*	*342.8 ± 61.3*		*309.0 ± 57.1*
Intromission frequency	326.2 ± 52.3	106.5 ± 34.9	417.7 ± 52.5	232.5 ± 49.1
	*391.5 ± 34.2*	*209.25 ± 33.7*		*310.0 ± 37.7*
Ejaculation frequency	51.0 ± 8.2	22.5 ± 8.5	50.2 ± 4.6	32.2 ± 7.1
	*61.2 ± 5.5*	*45.0 ± 10.9*		*43.0 ± 6.0*

*The values are based on either all tested animals or only on animals that copulated to ejaculation (written in italics). All RHA-experienced rats copulated to ejaculation, hence one value is reported. Values are expressed as Mean ± SEM. As no significant interactions between the rat line factor (RHA vs. RLA) and the sexual experience level factor (naïve vs. experienced) were found by analyzing the data with two way ANOVA as shown in Table 5, *post hoc* comparisons were not reported, as explained in the “Materials and Methods”, “Statistics” Subsection*.

**Table 5:** *F* values and significance levels of two-way ANOVAs performed on data reported in Table 4 by using the rat line (RHA vs. RLA) and the sexual experience level (naïve vs. experienced) as between subjects factors.

Parameter	*F* values			*df*
	Experience	Line	Experience × Line
DA	21.20***	8.22**	0.17	1, 1, 1, 44
	27.4***	6.04*	*2.76*	*1, 1, 1, 33*
DOPAC	0.16	67.90***	0.01	1, 1, 1, 44
	*0.20*	47.04***	*0.04*	*1, 1, 1, 33*
NA	1.50	62.72***	0.04	1, 1, 1, 44
	*0.53*	35.21***	*0.20*	*1, 1, 1, 33*
Non contact penile erection	4.96*	13.44***	0.00	1, 1, 1, 44
	*0.99*	10.60**	*0.00*	*1, 1, 1, 33*
Mount frequency	0.63	4.61*	0.00	1, 1, 1, 44
	*0.06*	*0.61*	*0.03*	*1, 1, 1, 33*
Intromission frequency	5.18*	17.97***	0.13	1, 1, 1, 44
	*1.90*	9.92**	*0.65*	*1, 1, 1, 33*
Ejaculation frequency	0.37	10.11**	0.51	1, 1, 1, 44
	*1.02*	*3.34*	*0.48*	*1, 1, 1, 33*

The values are based on either all tested animals or only on animals that copulated to ejaculation (written in italics). All RHA-experienced rats copulated to ejaculation, hence one value is reported. *P < 0.05; **P < 0.01; ***P < 0.001. Two way ANOVA of the data shown in Table 4.

**Table 6:** *F* values and significance levels of general factorial ANOVAs for repeated measures performed on the results shown in Figures 2 and 3 by using the rat line (L; RHA vs. RLA) and the sexual experience level (E) (naïve vs. experienced) as between subjects factors and time (T) (dialysate fractions) as within subjects factor.

Parameter	*F Values*							*df*
	L	E	T	L × E	L × T	E × T	L × E × T
DA#	6.01*	27.24***	27.50***	0.11	3.78***	3.52***	1.21	1, 1, 11, 1, 11, 11, 11, 484
	5.13*	32.13***	26.05***	*2.32*	3.10***	3.53***	*1.25*	*1, 1, 11, 1, 11, 11, 11, 363*
DOPAC#	68.54***	0.13	41.98***	0.00	2.32**	2.31**	0.89	1, 1, 11, 1, 11, 11, 11, 484
	46.94***	*0.16*	32.42***	*0.02*	*1.26*	2.92**	*0.81*	*1, 1, 11, 1, 11, 11, 11, 363*
NA#	59.95***	1.71	24.67***	0.00	5.20***	1.98*	0.72	1, 1, 11, 1, 11, 11, 11, 484
	35.36***	*0.91*	17.19***	*0.04*	5.58***	2.47**	*1.22*	*1, 1, 11, 1, 11, 11, 11, 363*
Non contact erections#	12.39**	4.77*	11.92**	0.03	0.51	0.06	0.00	1, 1, 1, 1, 1, 1, 1, 44
	10.11**	*0.83*	15.59***	*0.00*	*0.17*	*0.00*	*0.37*	*1, 1, 1, 1, 1, 1, 1, 33*
Mounts#	4.71*	1.32	14.10***	0.01	0.68	2.73*	1.90	1, 1, 4, 1, 4, 4, 4, 176
	*0.10*	*0.11*	13.05***	*0.24*	*0.56*	*2.38*	2.60*	*1, 1, 4, 1, 4, 4, 4, 132*
Intromissions#	11.24**	3.07	47.30***	0.81	2.05	2.83*	1.25	1, 1, 4, 1, 4, 4, 4, 176
	5.02*	*0.49*	53.74***	*2.38*	*0.70*	*0.79*	*1.33*	*1, 1, 4, 1, 4, 4, 4, 132*
Ejaculations#	10.93**	1.52	35.96***	0.33	2.42*	10.60***	1.10	1, 1, 4, 1, 4, 4, 4, 176
	*3.85*	*0.05*	40.06***	*0.43*	*1.36*	9.73***	*1.24*	*1, 1, 4, 1, 4, 4, 4, 132*

**Figure 3:**
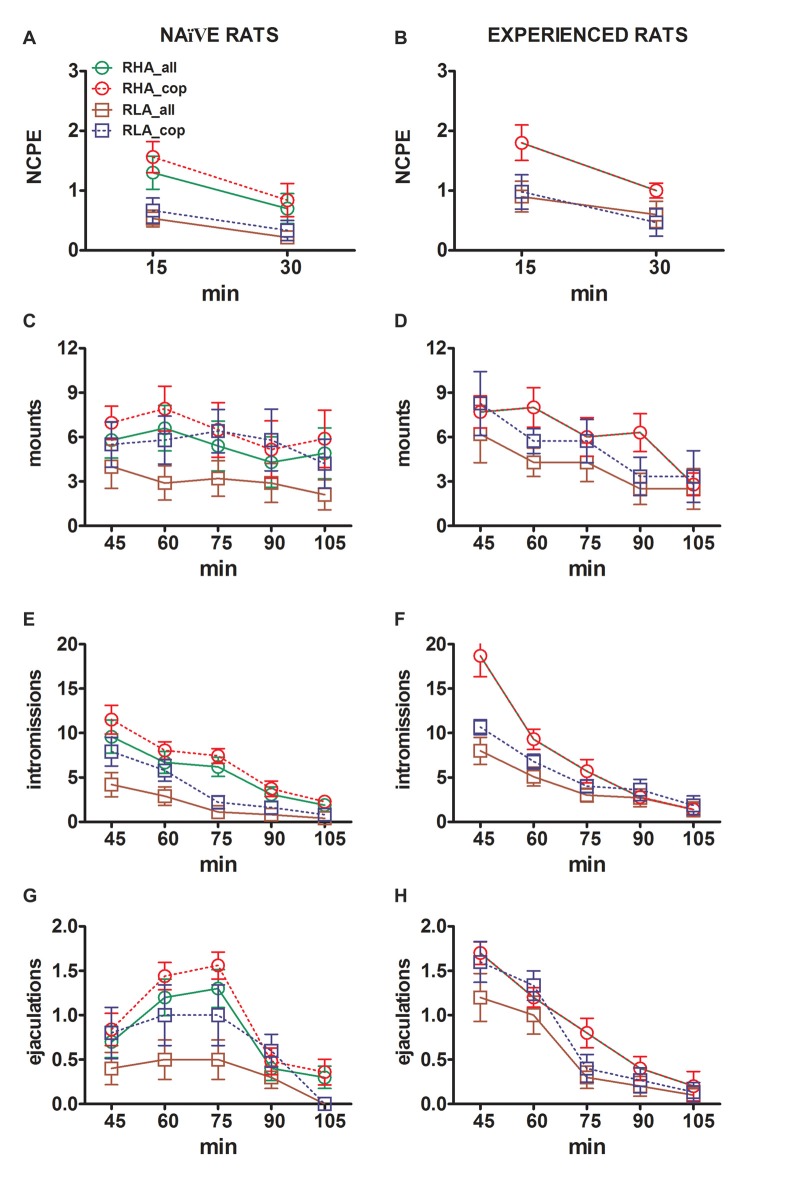
Changes in the number of non contact penile erections (NCPE), mounts, intromissions and ejaculations recorded from sexually naïve **(A,C,E,G)** and experienced RHA and RLA rats **(B,D,F,H)** during the microdialysis experiments reported in Figure 2. All the experimental conditions were identical to those described in the legend of Figure 2. Values are means ± SEM of the values obtained by all 12 rats per group (including those that were assigned full time scores; RHA = solid green lines, RLA = solid brown lines) or those that copulated to ejaculation only (RHA = dashed red lines; RLA = dashed blue lines). In experienced RHA rats all animals reached copulation and the values are identical. As no significant interactions between the rat line factor (RHA vs. RLA), the sexual experience level factor (naïve vs. experienced) and time were found by analyzing the data with two way ANOVAs as shown in Table 6, *post hoc* comparisons were not reported, as explained in the “Materials and Methods”, “Statistics” Subsection.

The values are based on either all tested animals or only on animals that copulated to ejaculation (written in italics). All RHA-experienced rats copulated to ejaculation, hence one value is reported. *P < 0.05; **P < 0.01; ***P < 0.001. ^#^Two way ANOVA of the data shown in Figures 2 and 3 after their logarithm transformation as explained in the “Materials and Methods”, “Statistics” Subsection.

## Results

### RHA and RLA Rats Show a Different Number of Non Contact Erections and Different Patterns of Copulatory Behavior

In line with earlier studies (Sanna et al., 2014a,b, 2015), a different number of sexually naïve male RHA and RLA rats became engaged in sexual activity when put together with a receptive female during the collection of dialysate aliquots from the mPFC by intracerebral microdialysis. Briefly, in this study 10 out of 12 sexually naïve male RHA rats (83%) copulated to ejaculation in their first copulatory test, against only 6 out of 12 naïve RLA rats (50%) during the microdialysis experiment. This difference was also found in sexually experienced male RHA and RLA rats (after five copulatory tests), although attenuated, with all 12 RHA rats reaching ejaculation against 9 out of 12 RLA rats during microdialysis. Always in agreement with previous studies, also in this study the two Roman rats lines exhibited different latencies and frequencies of non contact penile erections when exposed to an inaccessible receptive female as well as different patterns of copulatory behavior during sexual interaction. These differences were found either when considering the data of all experimental animals of a given group, regardless they copulated to ejaculation or not (e.g., by assigning full scores to those animals that did not show the behavior, that is rats which did not copulated to ejaculation) or when considering the data of the animals that did show the behavior of a given group only (e.g., animals which copulated to ejaculation; Table 1). Accordingly, statistical analyses of the values of non contact erections (NCPE) and of the copulatory parameters measured in the first series of copulatory activity by two-way ANOVAs revealed significant differences between the two rat lines either when considering all experimental rats or copulating rats only in the four experimental groups (see Table 2). In fact, when the data obtained from all experimental animals were considered, the number of non contact erections was higher and the NCPEL, ML, IL and PEI were significantly shorter in RHA rats compared to RLA rats. Further, the EF and the CE were higher, while the III was significantly shorter, in RHA rats compared to RLA rats. Some of the above differences between the two Roman rat lines tended to diminish or disappear with repeated copulatory tests. Nonetheless, some of these differences were still present after stabilization of sexual behavior by repeated sexual experience. Similar results were obtained when considering only the values of the animals of the four experimental groups that copulated to ejaculation, except for the EF (see Tables 1, 2).

### Basal Concentrations of Extracellular Dopamine, DOPAC and Noradrenaline in mPFC Dialysates from Sexually Naïve and Experienced RHA and RLA Rats

Under the present experimental conditions, the amounts of dopamine, DOPAC and NA in the dialysates obtained from the mPFC of all (e.g., regardless they copulated to ejaculation or not) sexually naïve RHA and RLA rats, were 2.32 pg and 2.11 pg for dopamine, 322.56 pg and 124.42 pg for DOPAC and 6.05 pg and 2.24 pg for NA, respectively, in 20 μl of dialysate. Similar amounts were measured in the dialysates obtained from the mPFC of sexually experienced RHA and RLA rats (dopamine: 3.73 pg and 3.33 pg, DOPAC: 353.62 pg and 133.32 pg, NA: 7.17 pg and 2.78 pg in RHA and RLA rats, respectively). These values indicate a concentration of ≅0.8–1.2 nM and ≅95–100 nM for extracellular dopamine and DOPAC, respectively, and ≅1.8–2.2 nM for extracellular NA, in the mPFC of RHA rats, and a concentration ≅0.8–1.2 nM and ≅35–40 nM for extracellular dopamine and DOPAC, respectively, and ≅0.6–0.9 nM for extracellular NA, in the mPFC of RLA rats (Table 3). The above values were obtained after a 2 h equilibration period of the dialysis buffer with the mPFC extracellular fluid. As the recovery of authentic dopamine, DOPAC and NA of the dialysis probes was estimated to be close to 20%, extracellular dopamine, DOPAC and NA concentrations may be estimated to be close to ≅4–5 nM in both lines for dopamine, ≅500 and ≅ 200 nM for DOPAC and ≅10 and ≅4 nM for NA in the mPFC of RHA and RLA rats, respectively. Factorial ANOVAs for repeated measures done on the above values after logarithm transformation, revealed significant differences in the basal levels of dopamine between sexually naïve and sexually experienced rats (*F*_(1,44,132)_ = 26.05, *P* < 0.001), and in the basal levels of DOPAC (*F*_(1,44,132)_ = 63.36, *P* < 0.001) and of NA (*F*_(1,44,132)_ = 42.14, *P* < 0.001) between RHA and RLA rats (last four samples collected before the introduction of the female in the small cage). Similar values were found when considering only rats which copulated to ejaculation during the microdialysis experiment (Table 3). Also in this case factorial ANOVAs for repeated measures done on the values after logarithm transformation revealed significant differences in the basal levels of dopamine between sexually naïve and sexually experienced rats (*F*_(1,33,99)_ = 26.97, *P* < 0.001), and in the basal levels of DOPAC (*F*_(1,33,99)_ = 42.95, *P* < 0.001) and of NA (*F*_(1,33,99)_ = 27.63, *P* < 0.001) between RHA and RLA rats.

### The Concentrations of Extracellular Dopamine, DOPAC and Noradrenaline in mPFC Dialysates from Sexually Naïve and Experienced RHA and RLA Rats Change Differentially during Sexual Activity

The presence of the inaccessible receptive female in the inner small cage and subsequent direct sexual interaction increased the concentrations of extracellular dopamine, DOPAC and NA in the mPFC dialysates obtained from both sexually naïve and experienced male RHA and RLA rats, although with significant differences between the two rat lines and level of experience conditions, either when considering all the experimental animals or only those that copulated to ejaculation (Figure 2). In fact, two-way ANOVAs of the AUCs of the concentrations of dopamine, DOPAC and NA, showed that the overall contents of dopamine, DOPAC and NA of the entire test, were significantly higher in RHA rats compared to RLA rats, and in sexually experienced compared to naïve rats (see Table 4 for AUCs values and Table 5 for *F* values and significance level). These differences were further confirmed by point to point factorial ANOVAs for repeated measures of dopamine, DOPAC and NA amounts of the two sexually naïve and experienced rat lines along the experiment, which revealed significant main effects of Line, Experience, Time and significant Line × Time and Experience × Time first order interactions (see Table 6 for *F* values and significance level).

#### The Concentrations of Extracellular Dopamine, DOPAC and Noradrenaline in mPFC Dialysates Obtained from Sexually Naïve RHA and RLA Rats Change Differentially during Sexual Activity

In sexually naïve RHA and RLA rats extracellular dopamine, but not DOPAC, increased first when put in the presence of the inaccessible female, while NA increased only in RHA rats. In naïve RHA rats, extracellular dopamine further increased in the first 15 min of sexual interaction as found with DOPAC and NA. The increments in extracellular dopamine, DOPAC and NA lasted for all the copulation time, with dopamine, DOPAC and NA reaching a peak value at 60 min, 75 min and 75 min, respectively, then extracellular dopamine and, to a lesser extent DOPAC and NA, decreased to values similar to the basal ones after removal of the female.

On the other hand, the first significant increase, and peak value, in extracellular dopamine, DOPAC and NA occurred in naïve RLA rats during the first 15 min of copulation. However, at variance from RHA rats, in RLA rats extracellular dopamine, DOPAC and NA tended to return to values similar to the basal ones within the first 30 min of direct interaction with the female (see Figures 2A,C,E).

Finally, during copulation, the increments in extracellular dopamine were larger and more persistent in sexually naïve RHA than RLA rats (see Figure 2A) whereas in the absence of the female their concentrations were similar, although not identical, across the two rat lines (see above and Table 3). Interestingly, although no difference was found between the two Roman lines in the percent increase of DOPAC and, to a lesser extent, NA concentrations during the presence of the receptive female, a difference of more than two fold in absolute values of DOPAC and NA concentrations was observed between the two rat lines both in basal conditions (see Table 3) and throughout the test with the receptive female (see Figures 2C,E).

#### The Concentrations of Extracellular Dopamine, DOPAC and Noradrenaline in mPFC Dialysates Obtained from Sexually Experienced RHA and RLA Rats Change Differentially during Sexual Activity

As found in sexually naïve RHA and RLA rats, the presence of the inaccessible female and subsequent direct sexual interaction led to an increase of extracellular dopamine and DOPAC in the mPFC dialysate obtained from sexually experienced male RHA and RLA rats. However, also in this case rat line-related differences were found, either when considering all the experimental animals or, to a lesser extent, only those that copulated to ejaculation (Figure 2). In sexually experienced RHA and RLA rats the first increase in extracellular dopamine occurred with the inaccessible female. Thereafter, dopamine concentrations increased during copulation in both rat lines. Dopamine concentrations reached their peak values after 45–60 min of copulation, and decreased slowly toward basal values at the end of the copulatory test (see Figure 2B). Similarly to sexually naïve rats, extracellular dopamine concentrations were higher in sexually experienced RHA rats compared to RLA rats mainly in the aliquots of dialysate collected during the central part of the copulation phase (after 30–45 min of copulation). DOPAC concentrations increased during the presence of the inaccessible female only in RHA rats, while it increased to a similar extent in both rat lines during copulation (peak values at 75–90 min), and tended to return to basal values at the end of the copulatory test (see Figure 2D). NA concentrations also increased in both sexually experienced rat lines after the introduction of the female reaching peak values at 90 min in RHA rats and at 45 min in RLA rats (see Figure 2F). At variance from dopamine (see above), similar difference were found in the increases of DOPAC and NA concentrations in sexually experienced rats from both lines when considering all animals vs. animals that reached ejaculation only. However, as found in sexually naïve rats, although the percent increases of DOPAC and NA concentrations were similar, significant differences in absolute values of DOPAC and NA were found between sexually experienced RHA and RLA rats throughout the test (i.e., before, during and after the presence of the receptive female), with RHA rats showing higher values than RLA rats (see Table 3 for basal values and Figures 2D,F for the values along entire test).

#### Sexual Experience Influences the Changes in Dopamine and Noradrenaline Concentrations in mPFC Dialysates Obtained from RHA and RLA Rats during Sexual Activity

A comparison of the concentrations of extracellular dopamine and NA in sexually naïve and experienced RHA and RLA rats showed that sexual experience changed the concentration of extracellular dopamine and, to a lesser extent, of NA in both rat lines, either when considering all the experimental animals or those that copulated to ejaculation only (Figures 2A,B,E,F). Accordingly, significant differences occurred in the basal values of dopamine between sexually naïve and experienced rats of both lines (see Table 3) and the dopamine values during copulatory activity were in general higher in sexually experienced RHA and RLA rats compared to their naïve counterparts (see Figure 2B). Moreover, in sexually experienced RHA rats, although the dopamine increase found during the presence of the inaccessible female followed the same temporal pattern seen in sexually naïve rats, differences in the temporal pattern were found during copulation. In fact, in this case, at variance from what observed in sexually naïve RHA rats (one main peak at 60 min), two main peak values were found, the first after 15 min and the second after 45 min of copulation. After this last increase, dopamine values tended to return to basal values (see Figure 2B).

A similar picture was found in sexually naïve vs. experienced RLA rats. In fact, similarly to what found in RHA rats, also in experienced RLA rats a temporal pattern characterized by three main increases in extracellular dopamine was found: the first with the inaccessible female and the other two during copulation, i.e., after 15 min and 60 min of copulation (see Figures 2A,B). In general, sexual experience seems to produce more long lasting changes in the temporal pattern of dopamine release in both rat lines when passing from the sexually naïve to the experienced condition.

As shown in Figures 2C–F, only small differences were detected in DOPAC and NA concentration between naïve and experienced rats of both lines. However, a not significant trend in extracellular NA toward higher values in experienced compared to naïve rats was observed in basal (before the introduction of the receptive female, see Table 3) and in the overall NA amounts, as revealed by AUCs values (see Table 4) calculated on extracellular concentrations obtained from the whole test (before and during the presence of the receptive female). Finally, the temporal pattern of NA concentrations was very similar when comparing experienced rats of both rat lines to their naïve counterparts, indicating that sexual experience can lead to a general increase in extracellular NA rather than to changes in the pattern of its increase (see Figures 2E,F).

### Changes in the Concentrations of Extracellular Dopamine and Noradrenaline in mPFC Dialysates Obtained from Sexually Naive and Experienced RHA and RLA Rats Occur Concomitantly with Changes in Sexual Behavior

The differences in the concentrations of extracellular dopamine, DOPAC and NA in the dialysates obtained from the mPFC found in both sexually naïve and experienced RHA and RLA rats during the test (Figure 2) occurred together with modifications in different sexual parameters measured during the two main phases of the experiments (i.e., female inaccessible and available to the male, respectively), which include, among others, non contact penile erections (Figures 3A,B), mounts (Figures 3C,D), intromissions (Figures 3E,F) and ejaculations (Figures 3G,H). These differences were found either when considering all the experimental animals or those that copulated to ejaculation only. In fact, a preliminary analysis of the AUCs values of these parameters by two-way ANOVAs (see Table 4 for AUCs values and Table 5 for *F* values and significance level) followed by a point to point analysis of the values of these parameters along the experiment by factorial ANOVA for repeated measures revealed significant main effects of Line, Experience, Time, significant first order Line × Time and Experience × Time interactions, and a significant second order interaction for mounts (see Table 6 for *F* values and significance level). Accordingly, in line with previous studies (Sanna et al., 2015): (i) sexually naïve RHA rats showed more non contact penile erections, mounts, intromissions and ejaculations than their RLA counterparts; (ii) sexually experienced RHA rats showed a higher number of non contact penile erections, intromissions and ejaculations than their RLA counterparts; and (iii) sexually experienced rats of both lines, and in particular RLA rats, showed more non contact penile erections, intromissions and ejaculations than their sexually naive counterparts. In fact, while in RHA rats the most part of the changes produced by sexual experience occurred in the time course rather than in the frequency of the behavioral parameters (e.g., experienced rats copulated mainly in the first half of the test, whereas naïve rats showed a similar copulatory trend throughout the test), in RLA rats a more marked general improvement of sexual behavior, mainly in sexual performance, was produced by sexual experience, as revealed by the changes of the values of copulatory parameters in experienced RLA rats compared with their naïve counterparts.

## Discussion

This study confirms and extends the findings of previous studies showing that RHA and RLA rats, which display markedly different coping styles in response to aversive conditions (Driscoll and Bättig, 1982; Giorgi et al., 2003a; Steimer and Driscoll, 2003), show distinct behavioral patterns in the presence of an inaccessible receptive female and during classical copulatory tests (Sanna et al., 2014a,b, 2015). Accordingly, also this study shows that sexually naïve RHA rats (never exposed to a receptive female before) exhibited higher sexual motivation and better sexual performance compared to sexually naïve RLA rats, e.g., RHA rats show higher number of non contact penile erections (pheromone-induced penile erections considered an index of sexual arousal; Sachs et al., 1994; Sachs, 2000) and different pro-sexual changes in several copulatory parameters as found in previous studies (Sanna et al., 2014a,b, 2015). These differences still persisted in both Roman rat lines after the acquisition of sexual experience, that is, after stabilization of sexual behavior with five expositions to and copulation tests with a receptive female. This study also shows for the first time that in both RHA and RLA rat lines, in either the naïve or experienced condition, non contact penile erections and sexual interaction, occurred concomitantly to an increase in the concentrations of extracellular dopamine (and its main metabolite DOPAC) and NA in the dialysates obtained by intracerebral microdialysis from the mPFC of both Roman rat lines. Interestingly, these increases in extracellular dopamine and NA in the mPFC appear to be related with differences either in the rat phenotype or in the level of sexual experience. Accordingly, as extensively shown in the Results section, both sexually naïve and experienced RHA rats displayed higher and long lasting increases in extracellular dopamine and NA when compared to their RLA counterparts in both the appetitive and consummatory phase of sexual behavior. Moreover, sexually experienced rats of both Roman lines displayed higher and long lasting increases in extracellular dopamine and NA in the mPFC in both phases of sexual behavior compared to their naïve counterparts. Finally, all the above differences in extracellular dopamine and NA in the mPFC were apparently related to differences in the behavioral parameters of sexual behavior, e.g., sexually experienced rats of both Roman lines displayed higher sexual motivation and better copulatory performances when compared to their naïve counterparts and sexually naïve and experienced RHA rats displayed higher sexual motivation and better copulatory performances when compared to their sexually naïve and experienced RLA counterparts.

As to the increases in extracellular dopamine found in the mPFC dialysate during sexual activity, the first increase occurred in the first 15 min after the introduction of the sexually receptive female in the mating apparatus, when the female was inaccessible to the male, which usually shows in this condition non contact penile erections. A second and larger increase in extracellular dopamine occurred during the first 15 min after the female was made accessible to the male for copulation. Interestingly, whereas in the naïve condition extracellular dopamine increased mainly in the first part of the test with the accessible female with a tendency to return to the basal values in the second part, in the sexually experienced condition dopamine remained elevated throughout the test reaching peak values after 45–60 min of copulation in both Roman rat lines.

Apart the above differences in extracellular dopamine during the different phases of sexual activity between RHA and RLA rats in the sexually naïve and experienced condition, this study also shows unexpected important and highly significant differences in extracellular DOPAC concentration between the two Roman rat lines before (i.e., basal values) and after the introduction of the receptive female in the mating apparatus. Accordingly, basal values of extracellular DOPAC in the mPFC dialysate were found in RHA rats to be about 2.6-fold higher than those found in RLA rats. This difference between RHA and RLA rats occurred and persisted in the sexually naïve and experienced conditions in spite of very modest differences in the basal values of extracellular dopamine in the mPFC dialysates obtained from the two Roman rat lines in these experimental conditions. Taken together, these differences might indicate a different dopamine turnover at the level of the mPFC between the two Roman rat lines. Accordingly, DOPAC concentrations usually reflect the amount of dopamine released and then recaptured by dopaminergic nerve terminals and converted to DOPAC by monoaminooxydase (MAO; Carlsson, 1975). Hence, the higher basal DOPAC concentrations found in RHA rats suggest that higher amounts of dopamine are released and recaptured by dopaminergic nerve endings in the mPFC of RHA rats, e.g., a higher basal activity of the mesocortical dopaminergic system of RHA rats when compared to RLA rats. A higher mesocortical dopaminergic tone in RHA rats than in RLA rats is also supported by the higher extracellular dopamine concentrations found during sexual activity in the mPFC dialysate from both sexually naïve and experienced RHA rats with respect to those of their RLA counterparts. In this regard, it is pertinent to recall that a higher dopaminergic tone is considered to be responsible, at least in part, of many of the different and even opposite behavioral traits present in RHA and RLA rats. Accordingly, RHA rats are active copers, highly impulsive, novelty and sensation seekers and are prone to intake and abuse several classes of drugs of addiction, while RLA rats are reactive copers, hyperemotional and are prone to develop depressive-like symptoms (Zeier et al., 1978; Giorgi et al., 1994, 2003b, 2007; Corda et al., 1997, 2014; Escorihuela et al., 1999; Steimer and Driscoll, 2003; Lecca et al., 2004; Giménez-Llort et al., 2005; Carrasco et al., 2008; Fattore et al., 2009; Moreno et al., 2010; Coppens et al., 2012; Díaz-Morán et al., 2012; Sabariego et al., 2013; Manzo et al., 2014a,b; Oliveras et al., 2015), including the higher sexual motivation and better copulatory performance of sexually naïve and experienced RHA rats vs. RHA rats (Sanna et al., 2015). The higher increase in the concentrations of extracellular dopamine found in the dialysate from the mPFC of sexually naïve and experienced RHA rats resembles the higher extracellular dopamine concentrations found in the dialysate from the nucleus accumbens of sexually naïve and experienced RHA rats with respect to their RLA counterparts (Sanna et al., 2015). However, at variance from the mPFC, the basal levels of extracellular dopamine and DOPAC in the dialysate from the nucleus accumbens of both sexually naïve and experienced RHA and RLA rats did not differ significantly (Sanna et al., 2015). Thus, the higher mesolimbic dopaminergic tone in the nucleus accumbens of sexually naïve and experienced RHA rats may be inferred only by the higher extracellular dopamine concentrations found in the dialysates from the nucleus accumbens of RHA rats when compared to RLA counterparts in the presence of the receptive female, and not by a different basal dopaminergic activity revealed by the higher and lower basal levels of DOPAC concentration in RHA rats and RLA rats, respectively, as found to occur in the mPFC in this study. This is also in line with the results of earlier reports showing that extracellular dopamine levels in the dialysate from the mPFC of RHA rats were found higher than those of RLA rats, while similar dopamine levels were found in the dialysate from the shell of the nucleus accumbens of both RHA and RLA rats under different experimental conditions (D’Angio et al., 1988; Scatton et al., 1988; Willig et al., 1991; Giorgi et al., 2003a, 2007). In spite of the above differences between mPFC and the nucleus accumbens, sexual experience seems to produce in the mPFC longer lasting increases in the temporal pattern of dopamine release in both Roman rat lines when passing from the sexually naïve to the experienced condition, although such increases were usually higher in RHA rats than RLA rats. This is somewhat different from what found in the nucleus accumbens, where sexual experience tended to shift extracellular dopamine increases to the first part of the copulatory test in RHA rats, while increased it throughout the entire copulation test in RLA rats (Sanna et al., 2015).

To our knowledge, this study also shows for the first time that not only extracellular dopamine, but also extracellular NA increases during sexual activity in the dialysate from the mPFC of both RHA and RLA rats, as found with dopamine, but also in this case with differences related to the Roman rat line and to sexual experience. In particular, at variance from basal dopamine levels that were found very similar in the dialysate from the mPFC of both RHA and RLA rats, the basal levels of extracellular NA in the dialysate from the mPFC of RHA rats were significantly higher (about 2,5-fold) than those of RLA rats. The reasons for these differences are unknown, but as discussed above for dopamine and DOPAC, these results may indicate that a higher basal noradrenergic tone exists at least in the mPFC cortex of RHA rats when compared to RLA rats. Such a higher noradrenergic tone may also be involved in the different and often opposite behavioral traits present in RHA and RLA rats, as discussed above for dopamine. Further support for a higher noradrenergic tone in RHA rats than in RLA rats in the mPFC comes from the results showing that the differences in basal extracellular NA levels persisted also during sexual activity in both sexually naïve and experienced conditions, e.g., extracellular NA levels increased in the mPFC dialysate in RHA rats when exposed to the inaccessible female and during all the copulation period much more than in RLA rats, in which NA levels increased only in the first 15 min of the copulation period, after which NA levels returned back to basal values. These changes in extracellular NA levels were seen in both the sexually naïve and experienced condition, but the increases in NA levels were higher in sexually experienced RHA and RLA rats compared to their sexually naïve counterparts. Thus, sexual experience apparently induces a further potentiation of the noradrenergic activity in the mPFC of RHA rats and, to a lesser extent, of RLA rats, as already discussed for dopamine. The concomitant higher noradrenergic and dopaminergic tones in the mPFC of RHA rats when compared to RLA rats deserve some comment. In fact, it was reported that noradrenergic activity in the mPFC can affect dopamine release through different mechanisms (for example see Carboni et al., 1990, 2006; Gresch et al., 1995; Westernik et al., 1998 and references therein). One of these is related to the activity of the NET, which was shown to bind dopamine with an affinity even higher to that for NA in synaptosomes obtained from homogenates of the PFC (Horn, 1973). If this were to occur in the mPFC *in vivo*, dopamine released in the mPFC could be recaptured not only by dopaminergic but also by noradrenergic nerve terminals at a speed even higher than that of NA. This could cause in turn an increase in dopamine release from dopaminergic terminals (e.g., an increased dopaminergic tone) to compensate the amount of dopamine removed from the synaptic cleft; thus, the higher noradrenergic tone found in RHA rats could contribute, at least in part, to the higher dopaminergic tone found in the mPFC of RHA rats when compared to RLA rats. Further studies are required to verify this possibility. Notably, the difference in noradrenergic activity identified in this study in the mPFC between RHA and RLA rats might also play a role in their different response to antidepressant treatment. In fact, RHA rats are considered a model of resilience and RLA rats a model of vulnerability to depression. Accordingly, acute or chronic treatment with antidepressants like desipramine, fluoxetine and chlorimipramine, all significantly improved the behavioral responses in the forced swimming test in RLA rats without affecting the responses of RHA rats (Piras et al., 2010, 2014).

Finally, this study confirms and extends previous findings showing that the mPFC is involved in the control of sexual behavior, although the exact role of this brain area in sexual behavior is far from being clear (Fernández-Guasti et al., 1994; Agmo and Villalpando, 1995; Agmo et al., 1995; Hernández-Gonzáles et al., 1998, 2007; Kakeyama et al., 2003; Balfour et al., 2006; Afonso et al., 2007; Davis et al., 2010; Febo, 2011). Recently, selective cell firing in the mPFC during approaching behaviors of a male rat toward an inaccessible sexually receptive female was shown using a single cell firing recording paradigm (Febo, 2011). Interestingly, neurons which did not respond during the first exposition of the male to the inaccessible receptive female, become active during the second exposition, as if the previous experience was able to induce plastic changes leading to variation in the behavioral responses. Nonetheless, lesions with ibotenic acid of the mPFC were found unable to alter sexual behavior of male rats with a sexually receptive female, nor the expression of conditioned place preference for sexual reward. However, the ability to form conditioned aversion toward sexual activity when paired with aversive stimuli was found completely abolished in these lesioned animals (Davis et al., 2010). This suggests that mPFC activation during sexual behavior plays a role in the integration of external and internal information for the execution and control of goal-directed behaviors rather than in the expression of innate responses to natural reinforcers (see Goto and Grace, 2005). More important for this work, several studies have shown that brain areas involved in sexual motivation and sexual behavior such as the nucleus accumbens, ventral tegmental area, medial preoptic area, bed nucleus of the stria terminalis, basolateral amygdala and parvocellular subparafascicular thalamic nucleus receive inputs from the mPFC during sexual activity (see Balfour et al., 2006). Many of these brain areas participate in a complex neural circuit involved in the control of sexual behavior, from sexual motivation and reward to sexual performance. This circuit includes oxytocinergic neurons that originate in the PVN of the hypothalamus (PVN) and project to the ventral tegmental area, nucleus accumbens, hippocampus, amygdala, PFC, bed nucleus of the stria terminalis, medulla oblongata and spinal cord, mesolimbic/mesocortical dopaminergic neurons projecting from the ventral tegmental area to the nucleus accumbens and PFC, incertohypothalamic dopaminergic neurons and glutamatergic neurons that participate at local and system level in several areas of the circuit (see Melis and Argiolas, 1995, 2011; Melis et al., 2003, 2007, 2009, 2010; Succu et al., 2007, 2008, 2011). Thus, the increase of extracellular dopamine (but also of NA) in the dialysates from the mPFC seen during sexual activity might indicate that the mPFC is an active part of this circuit, that is, mesocortical dopaminergic neurons participate in the control of sexual activity together with noradrenergic, mesolimbic dopaminergic, oxytocinergic and glutamatergic neurons, although it seems not strictly necessary for the expression of sexual behavior (Davis et al., 2010). In line with this hypothesis, this study shows that the differences in dopamine and NA release in the mPFC during sexual activity occurred concomitantly with differences in the number of non contact penile erections and changes in copulatory parameters, with a higher release of dopamine and NA during the appetitive and consummatory phases related to higher sexual motivation and better copulatory performances in both Roman rat lines and in both the sexually naïve and experienced conditions.

The discussion given above is based mainly on the well known increase of extracellular dopamine that occurs in the nucleus accumbens of male rats during sexual activity (Pfaus et al., 1990; Pleim et al., 1990; Pfaus and Phillips, 1991; Damsma et al., 1992; Wenkstern et al., 1993). However dopamine and other neurotransmitters might be released in the mPFC (and in the nucleus accumbens) in other experimental conditions, such as during stress or coping with it (Thierry et al., 1976; Fadda et al., 1978), which are not strictly related to sexual behavior. Thus, one may argue that the dopamine increase found in extracellular dopamine in the mPFC (this study) or in the nucleus accumbens (Sanna et al., 2015), is related more to the stress secondary to, or to the coping with the presence of and/or interaction with the sexually receptive female rather than to sexual activity. This might have some relevance in RHA and RLA rats, which show markedly different coping styles and different hypothalamic-pituitary- adrenal axis activation in response to aversive conditions (RLA rats show an activation of this axis much higher than that of RHA rats; see Carrasco et al., 2008; Díaz-Morán et al., 2012). This also raises the possibility that stress hormones (i.e., corticosteroids) may influence dopaminergic activity in the mPFC and other brain areas as well. Indeed RLA rats are reactive copers and show hyperemotional behavior characterized by hypomotility and freezing, while RHA rats show a proactive coping behavior aimed at gaining control over the stressor (Driscoll and Bättig, 1982; Willig et al., 1991; Escorihuela et al., 1999; Steimer and Driscoll, 2003; Giorgi et al., 2007). Although it is impossible to rule out completely that this may occur in sexually naïve rats (which never interacted with a receptive female before) and in sexually naïve RLA rats in particular (see above), this is unlikely. In our experimental conditions all possible is done to avoid all kind of stress (see “Materials and Methods” Section) and experiments are organized in such a way that males do not interact immediately with the female, but only after 30 min of exposition to her, during which the measured increase in dopamine is much lower than that occurring during sexual interaction. If the dopamine increase found in the mPFC (and in the nucleus accumbens) of Roman rats (sexually naïve RHA and RLA rats have basal levels of extracellular dopamine in the dialysate from the mPFC and the nucleus accumbens, very similar) were really secondary to the stress due to, or the coping with the presence of/interaction with the female: (i) the dopamine increase would have been found much higher in the first period of time when the males are put in the presence of the female and not later, as found during copulation; and (ii) dopamine increase would be expected to disappear or to be strongly reduced after acquisition of a stable sexual activity, that is, when learning processes (including coping to novelty) have been completed and play only a minor role in the execution of sexual activity, while in contrast it continues to occur when sexual activity is repeated. In other words, extracellular dopamine in the mPFC (and in the nucleus accumbens) always increases during copulation, even after sexual behavior has been learned. Further studies are required to clarify the functional role of this increase in dopamine activity in the mPFC (and in the nucleus accumbens; see "Introduction and References" Section therein).

In conclusion, this study shows for the first time that the presence of, and even more, the interaction with a sexually receptive female leads to an increase of extracellular dopamine and NA in the dialysate from the mPFC of sexually naïve (never exposed to sexual stimuli) and sexually experienced (which underwent five preliminary copulation tests and show stable sexual performances) RHA and RLA rats, which display markedly different coping styles in response to aversive conditions and different patterns of copulatory behavior with a sexually receptive female as well. Accordingly, dopamine and NA concentrations were found higher in the dialysate from sexually naïve and experienced RHA rats when compared to their RLA counterparts during both the anticipatory and consummatory phases of sexual activity. These results may be due in part to a dopaminergic and noradrenergic tone in the mPFC of RHA rats higher than that of RLA rats, as reported for dopamine in the nucleus accumbens (Sanna et al., 2015). Moreover, extracellular mPFC dopamine and NA were also found higher in sexually experienced RHA and, to a lesser extent, RLA rats compared to their sexually naïve counterparts in basal conditions as well as during the appetitive and consummatory phases of sexual behavior. This suggests that sexual experience induces plastic processes that further potentiate dopamine and NA neurotransmission in the mPFC, as found for dopamine in the nucleus accumbens of both Roman rat lines (Sanna et al., 2015). The above differences in dopaminergic and noradrenergic neurotransmission in the mPFC of the two Roman lines may play a role not only in the different patterns of sexual behavior but also in the different and often opposite behavioral traits that characterize these two rat lines.

## Author Contributions

FS, AA, MRM, OG and MGC designed the project. FS and JB designed, performed and analyzed the data from sexual behavior and microdialysis experiments. MAP, OG and MGC selected and bred Roman rats. FS, AA, MRM, OG and MGC supervised the study. FS, AA, MRM, OG and MGC wrote the manuscript. All authors discussed the results and commented the manuscript.

## Conflict of Interest Statement

The authors declare that the research was conducted in the absence of any commercial or financial relationships that could be construed as a potential conflict of interest.
